# Two Novel Techniques to Screen *Abies* Seedlings for Resistance to the Balsam Woolly Adelgid, *Adelges piceae*

**DOI:** 10.1673/031.011.15801

**Published:** 2011-11-17

**Authors:** Leslie Newton, John Frampton, John Monahan, Barry Goldfarb, Fred Hain

**Affiliations:** ^1^ Department of Entomology, North Carolina State University, Box 7626, Raleigh, NC 27695; ^2^ Department of Forestry and Environmental Resources, North Carolina State University, Box 8008, Raleigh, NC 27695; ^3^ department of Statistics, North Carolina State University, Box 8203, Raleigh, NC 27695

**Keywords:** *Abies fraseri*, artificial infestation, excised branches, Fraser fir, host resistance

## Abstract

Since its introduction into the Southern Appalachians in the 1950s, the balsam woolly adelgid, *Adelges piceae* Ratzeburg (Hemiptera: Adelgidae), has devastated native populations of Fraser fir, *Abies fraseri* (Pursh) Poir. (Pinales: Pinaceae), and has become a major pest in Christmas tree plantations requiring expensive chemical treatments. *Adelges piceae*—resistant Fraser fir trees would lessen costs for the Christmas tree industry and assist in the restoration of native stands. Resistance screening is an important step in this process. Here, four studies directed toward the development of time— and cost—efficient techniques for screening are reported. In the first study, three methods to artificially infest seedlings of different ages were evaluated in a shade—covered greenhouse. Two—year—old seedlings had much lower infestation levels than 7 year—old seedlings. Placing infested bark at the base of the seedling was less effective than tying infested bark to the seedling or suspending infested bolts above the seedling. Although the two latter techniques resulted in similar densities on the seedlings, they each have positive and negative considerations. Attaching bark to uninfested trees is effective, but very time consuming. The suspended bolt method mimics natural infestation and is more economical than attaching bark, but care must be taken to ensure an even distribution of crawlers falling onto the seedlings. The second study focused on the density and distribution of crawlers falling from suspended bolts onto paper gridded into 7.6 × 7.6 cm cells. Crawler density in a 30 cm band under and to each side of the suspended bolt ranged from 400 to over 3000 crawlers per cell (1 to 55 crawlers per cm^2^). In the third study, excised branches from 4 year—old *A. fraseri* and *A. vetchii* seedlings were artificially infested with *A. piceae* to determine whether this technique may be useful for early resistance screening. The excised *A. fraseri* branches supported complete adelgid development (crawler to egg—laying adult), and very little adelgid development occurred on *A. vetchii* branches. The fourth study compared infestation levels and gouting response on excised versus intact branches of 4 year—old *A. fraseri* seedlings from three different seed sources, and excised branches from 4 year—old and 25 year—old trees. There were no differences in infestation levels between excised versus intact branches nor in very young versus mature trees; gouting response was observed only on intact branches.

## Introduction

The balsam woolly adelgid, *Adelges piceae* Ratzeburg (Hemiptera: Adelgidae), a tiny, piercing—sucking insect specific to the genus *Abies*, is a pest native to central Europe that has been introduced to North America. Since its introduction into the Southern Appalachians in 1955 (Amman 1966), *A. piceae* has contributed to the decline of Fraser fir, *Abies fraseri* (Pursh) Poir. (Pinales: Pinaceae), throughout its native range, and has become a major pest in Fraser fir Christmas tree plantations. There are ∼ 50 million Fraser fir trees growing on over 10,000 hectacres in North Carolina alone, providing annual cash receipts of well over $100 million to the Christmas tree industry (Sidebottom 2008). Chemical insecticides are the only effective means for controlling this pest, and treatments exceed $1.5 million per year (Potter et al. 2005). The frequent need to spray plantations for *A. piceae*, in addition to being expensive for growers, minimizes the effectiveness of integrated pest management (IPM) practices. Insecticides reduce populations of natural enemies, causing otherwise minor pests (e.g., spruce spider mites, *Oligonychus ununguis*) to increase dramatically (Sidebottom 2009), requiring more chemical input into the system.

In North America, *A. piceae* reproduces through parthenogenesis and completes two or more generations per year (Balch 1952; Arthur and Hain 1984). Life stages consist of eggs, three instars, and adults. The early phase of the first instar (crawler) is the only motile stage, and it is primarily wind or gravity dispersed. Feeding sites are chosen for accessibility to parenchyma cells (Balch 1952). The crawler inserts its stylets into the bark, and the adelgid remains in that location for the remainder of its life. White, ribbon—like threads are secreted from wax glands (Balch 1952), eventually covering the adelgid and giving the appearance of cottony or woolly tufts. This ‘woolly mass’ is the most obvious sign of infestation.

Susceptibility to *A. piceae* within the genus *Abies* varies considerably. Firs native to North America, e.g., subalpine fir *Abies lasiocarpa*, balsam fir *Abies balsamea*, and Fraser fir, are highly susceptible, while firs native to central Europe e.g., European silver fir *Abies alba*, are able to tolerate infestation for years with relatively few ill effects (Varty 1956; Mitchell 1966). Some Asian species e.g., Veitch fir *Abies veitchii* appear to be almost immune to attack (Hall et al. 1971). Host resistance mechanisms, while not yet fully understood, appear to include a thick outer bark (Balch 1952; Varty 1956), rapid formation of secondary periderm around the feeding site (Amman and Fedde 1971, Hollingsworth and Hain 1992), and accumulation (constitutive or induced) of chemicals (e.g., monoterpenes or sesquiterpenoids) at the site of attack that may interfere with adelgid development (Hain et al. 1991; Balakshin et al. 2005). Mature trees in natural stands appear to be more susceptible to *A. piceae* attack (Mitchell 1966), although saplings (5.1 cm diameter at breast height) of various fir species have been observed with infestations (Harris 1973), and even the youngest trees in Fraser fir Christmas tree plantations are vulnerable to infestation.

Although *A. piceae* infestations often result in the death of susceptible host trees, there are reports of full recovery from severe infestations in some species such as noble and white firs *Abies procera* and *Abies concolor*, respectively (Mitchell 1966), European silver fir (Kloft 1957), West Virginia Canaan fir *Abies balsamea* var. *phanerolepis*, and even Fraser fir (Hain et al. 1991). This, coupled with the presence of numerous old—growth survivor Fraser firs in the high elevation forests of the Great Smoky Mountains National Park (Kloster 2001; Johnson et al. 2005), suggests there may be some element of resistance or tolerance within the Fraser fir. The predominant hypothesis under evaluation is that there is a genetic basis for host resistance in firs.

The utilization of genetically resistant Fraser fir planting stock for Christmas tree production would be a relatively inexpensive solution to a difficult pest problem and minimize adverse effects from the pest and related management strategies. In addition, resistant trees could be utilized in the restoration of native Fraser fir stands, which exist only as mountain top populations in Virginia, Tennessee, and North Carolina. Over 95% of mature trees were killed after the initial infestation of *A. piceae* in the 1950s, and although many of these stands have recovered from the initial wave of devastation through regeneration from wild seeds, the endemic population is shrinking and their long—term prospects are uncertain. Outplantings of *A. piceae—resistant* Fraser fir could increase the likelihood of the continued survival of this ecologically and recreationally important ecosystem.

Resistance screening is an important step in the process of discovering and breeding resistance, requiring an effective, time— and cost—efficient technique for infesting large numbers of trees at the same time and under the same conditions. The most commonly reported infestation technique involves cutting bark pieces with egg masses from infested field trees and attaching the bark pieces to the stems of uninfested trees or seedlings (with the egg masses facing the new host) (Tunnock and Rudinsky 1959; Carrow and Graham 1968; Carrow 1971; Hollingsworth and Hain 1994a). This is a reliable, yet time— consuming, technique. Prior artificial infestations of Fraser fir seedlings in a laboratory or greenhouse setting have met with limited success, possibly because temperatures were too high or too constant, or because too few eggs and crawlers were utilized in the studies (Hollingsworth 1990; Hollingsworth and Hain 1994a, 1994b). Balch (1952) suggested that 30 eggs may produce one adult, 500 eggs 10 adults, and 1200 eggs may result in a moderately heavy infestation. Settled neosistentes (first instar larvae) suffer high mortality; most crawlers will settle and enter into a period of rest, but most never come out of that stage to continue their development. Optimum temperatures reported for maximizing development and minimizing mortality on *A. grandis* are cycles between 13 °C and 24 °C (Atkins 1972). Our preliminary studies found that both constant (17 °C) and ramping (13—24 °C) temperatures and attaching bark with 20 to 180 eggs resulted in successful infestation of 1 to 6 year—old Fraser fir seedlings (Newton, unpublished data). These studies proved that Fraser fir seedlings of various ages could be successfully infested artificially with *A. piceae*, but the amount of time required to cut and attach bark pieces to each seedling was felt to be excessive for utilization in resistance screening trials. Thus, a new technique was developed that mimics natural infestation and reduces implementation time. This new technique involves suspending bolts of heavily infested Fraser fir over seedlings, allowing the hatching crawlers to fall onto the uninfested trees below. This technique was tested on 2 and 7 year—old trees and is described and reported in Studies I and II below.

Host resistance screening trails are often accomplished with young trees grown from seed (Nielsen et al. 2002; Alfaro et al. 2008) or as clonal material (Foster and Anderson 1989). These approaches require three or more years before the material can be utilized in a screening trial. The ability to screen trees of any age, either from existing grafted clone banks, plantations, or natural stands, would enable researchers to test for resistance using a wider range of genetic material in a more time—efficient manner. Fraser fir is well known for maintaining freshness after harvest, and exhibits excellent needle retention even 35 days post—harvest (Bates et al. 2004). With this in mind, a new technique for artificially infesting excised branch tips with *A. piceae* has been developed and is being tested for its potential to serve as a means to pre—screen Fraser fir trees for resistance trials. The early work conducted with excised branches is reported in Studies III and IV.

## Materials and Methods

### Study I: Comparison of three artificial infestation techniques

Two ages of Fraser fir were utilized, 2 and 7 year—olds, both from the Roan Mountain seed source. The 2 year-old bareroot seedlings were obtained from the North Carolina Division of Forest Resources in May 2006 and transplanted into 7.6 × 7.6 × 22.9 cm tree pots. The 7 year—old trees were 3—1 Fraser fir seedlings obtained from the North Carolina Division of Forest Resources in 2003, transplanted into 11.4 liter pots, and grown at the North Carolina State University Horticultural Field Lab in Raleigh until June 2006, at which time they were transported to Ashe County, North Carolina. Potting medium consisted of mulched pine bark. The trees were fertilized with Osmocote (Scotts Co., www.scotts.com) 19-5-8 slow—release formula; nitrogen sources in this formula are provided equally as nitrate, ammonium nitrate, and urea. An impact—head sprinkler system was installed in the greenhouse to provide irrigation and the screen over the frame allowed rain as well.

The study was conducted at the North Carolina Department of Agriculture Upper Mountain Research Station in a shade— covered 6.1 m × 12.2 m greenhouse. The greenhouse was divided into four blocks, each containing six treatment plots. Each plot measured 0.9 m × 1.2 m × 1.2 m, with the perimeter of each surrounded by organza cloth supported by 1.3 cm diameter PVC pipe to provide a barrier to prevent dispersal of crawlers between treatments. Each plot contained 12 potted Fraser firs, consisting of an equal number of 2 and 7 year—old seedlings arranged so that their crowns were level. One seedling of each age served as an uninfested control for two of the treatments. Seasonal effect was examined by infesting three of the treatment plots in the summer and three in the fall, with each plot receiving one of three infestation techniques.

Two of the three infestation techniques used pieces of bark containing *A. piceae* egg masses taken from infested *A. fraseri* trees cut from an abandoned Christmas tree plantation in Ashe County. The bark pieces averaged 10 eggs per woolly mass. Sufficient bark pieces were attached to total 50 egg masses on the 7 year—old trees and 10 egg masses on the 2 year—old trees. One treatment (Attach Bark) consisted of attaching the bark pieces along the stems of the uninfested seedlings with horticultural ties. Another treatment (Place Bark) placed the infested bark pieces at the base of the uninfested seedlings. The third treatment (Suspend Bolt) suspended 1.2 m long bolts of BWA—infested Fraser fir over uninfested seedlings so that hatching crawlers would fall off the bolt and onto the seedlings below.

The infestation treatments were applied to the seedlings three times, for a duration of one week each time. During the summer season the fir seedlings were infested on 5 July 2006, 12 July 2006, and 19 July 2006; during the fall season the seedlings were infested on 20 September 2006, 27 September 2006, and 11 October 2006. The total number of eggs using the bark pieces was 300 and 1500, respectively, for each 2 and 7 year—old seedling. For the Suspend Bolt treatment, one bolt was suspended on each of the three dates: the first time across the center of the treatment plot, the second diagonally from corner to corner, and the third diagonally in the opposite direction.

The infestations were allowed to develop through winter dormancy and into the spring. The greenhouse was covered with white plastic between December and April. The study was dismantled in June 2007; each tree was cut at the base, placed individually in polyethylene bags, transported to North Carolina State University, and stored in a cooler at 4 °C pending assessment. Each tree was assessed and measurements were taken for height, diameter (2.5 cm above base), and the total number of *A. piceae* woolly masses per tree. There were 288 trees utilized in this study, including 256 treatment trees and 32 controls. The Attach Bark and Place Bark treatments included untreated controls; the Suspend Bolt treatment did not include controls. Because of a treatment application problem, all 12 trees from Block 4, Treatment 2 (Place Bark), Season 2 (fall) were deleted from the analysis. Another 101 trees were lost through death or mold problems in storage. A total of 157 treatment trees and 18 controls were analyzed.

### Study II: Density distribution of crawlers dropping from bolts

In July 2007, logs from heavily infested Fraser fir trees in an abandoned Christmas tree plantation in Ashe County were cut and transported back to North Carolina State University. The following day, two logs (10.2 cm in diameter and 1.2 m or 1.5 m length) were suspended over paper gridded into 7.6 × 7.6 cm cells, and sprayed with Tanglefoot® (Contech Enterprises, www.contech-inc.com) to trap the falling crawlers. The logs were suspended 0.91 m above the grids. One log was suspended in a relatively windless environment in the laboratory and one was suspended in an air—conditioned space within a greenhouse.

The logs were left suspended for five days. On the sixth day, each grid was covered with clear transparencies and the grids cut into squares (7.6 × 7.6 cm cells) for assessment. A subsampling scheme was developed that consisted of selecting cells representative of all levels of crawler densities and counting the crawlers under a microscope. A total of 18 samples were counted which had crawler densities of 24, 51, 73, 115, 243, 330, 418, 550…3035. For the remaining cells (174 and 240 for the 2 logs, respectively), crawler densities were estimated by finding the most similar cell within the counted samples and using that known quantity to approximate the number of crawlers.

### Study III: Infestation of excised Fraser fir and Veitch fir branches from 4 year—old seedlings

In May 2007, four branches were taken from each of three trees of Fraser and Veitch firs. The excised branches (cuttings) were placed individually in glass tubes filled with wet sand. Bark discs (8–10 egg masses, each with 10–15 eggs) were taken from *A. piceae*— infested Fraser fir and attached to each branch with wire. The samples were split into two groups (randomized in trays with six cuttings per species per group) and stored in two incubators, both held at a constant temperature of 17 °C. Fluorescent grow lights were placed in each incubator and a timer installed to provide light for the cuttings (long days: 16:8 L:D). Humidity was not controlled. The cuttings were watered twice a week and assessed for *A. piceae* development four, seven, and nine weeks following infestation. Measured variables included the numbers of crawlers, settled instars, adults, and eggs. The study included 12 branches per species.

### Study IV: Infestation of intact and excised branches of 4 year—old Fraser fir seedlings and excised branches of 25—year—old Fraser fir trees.

This study included 4 year—old Fraser fir from three seed sources (Richland Balsam, Mount Mitchell, and Roan Mountain) and 25—year— old Fraser fir (Roan Mountain seed source). The seedlings were grown in a greenhouse in Raleigh, NC, and the mature trees were growing in a plantation in Ashe County, NC. The design included five seedlings per seed source for the 4 year—old trees and branches from five mature (25—year—old) trees. In September 2007, 3 branches were cut from each 4 year—old seedling and the mature field trees. The excised branches were placed into sand-filled plastic centrifuge tubes, randomized in trays, and placed into plastic tubs. To keep the cuttings hydrated, the tubes and trays had small holes cut in the bottom, and water was added to completely saturate the sand. The trees were watered three times per week and water was added to the tub with cuttings three times per week. Water was also squirted into each centrifuge tube to keep the sand moist. The seedlings and excised branches were held at a constant temperature of 17 °C. Humidity was not controlled and artificial lighting (long days) was provided with fluorescent grow lights. Excised branches from each age class and two to three intact branches from the 4 year—old seedlings were infested with *A. piceae*. Bark discs (with 8—10 egg masses, each with 10—15 eggs and a few crawlers) were taken from *A. piceae*— infested Fraser fir and attached to the branches with wire. The discs were attached either at the branch node or at the tip of the branch (near the bud). Infestation levels were assessed after ten weeks, represented by the numbers of settled first instars, adults, and eggs. The condition (health or quality) of each branch specimen was assessed (poor (chlorotic or brown), fair (somewhat chlorotic), or good (deep green)), and whether or not gouting (swollen areas at the site of infestation) had taken place (yes/no). Also noted was the location that the crawlers settled in relation to the placement of the infested bark discs. The study included a total of 45 excised and 39 intact branches from the 4 year—old seedlings and 15 excised branches from the mature trees.

### Statistical analysis

Responses in each of the four studies were tested for normality (Shapiro-Wilk) using the Univariate procedure (PROC UNIVARIATE) of SAS version 9.1 (SAS Institute 2003). Transformations were applied as necessary to bring the distributions closer to normality and to make the variances more homogeneous. For each study, an analysis of variance was performed using the General Linear Model procedure (PROC GLM), and pairwise comparisons were analyzed by the Tukey-Kramer Multiple Comparison Test (SAS Institute 2003) where appropriate. Data are presented as least squares means ±standard error; if a transformation was utilized, the results were back—calculated for reporting. Details specific to each study are provided as follows:

**Study I**. The GLM procedure incorporated the following effects: block, infestation technique, age, season, technique × age interaction, and season × age interaction. Block was considered a random effect; all others were fixed effects. A logarithmic transformation (log * (1 + x)) was applied to the response (number of woolly masses containing adults with egg masses). An additional ANOVA was performed comparing the untreated controls with the treated trees from the Attach Bark and Place Bark treatments.

**Study II**. Data for each cell were entered into a spreadsheet and contour plots showing crawler density were created utilizing SigmaPlot for Windows version 10.0 (Systat Software Inc., www.systat.com). The two repetitions were assessed separately.

**Study III**. The main effect was species (Fraser fir versus Veitch fir).

**Study IV**. Two analyses were performed, the first (to compare excised and intact branches) with the 4 year—old trees from different seed sources. The main effects were form (intact versus excised branches) and seed source (Richland Balsam, Mount Mitchell, and Roan Mountain). The first instar counts were transformed (square root (x)) for analysis and back—transformed for reporting. The second analysis (to compare young seedlings with mature trees) was performed with excised branches from the 4—year—old seedlings (Roan Mountain only) and the 25 year—old trees (also Roan Mountain). The main effect was age.

## Results

### Study I: Comparison of three artificial infestation techniques

Attaching infested bark pieces and suspending infested bolts were equally effective, resulting in higher numbers of *A. piceae* infestations than placing bark at the base of tree (Table 1). All treatment effects included in the general linear model were significant (*p* < 0.01) (Table 2). There were significant infestation technique interactions by age (*p* < 0.01) and season (*p* < 0.05). The data may best be understood by examining the means for these interactions (Table 3). The 2 year-old seedlings averaged < 1 woolly mass per seedling, and the 7 year-old trees averaged 30.6 woolly masses per tree. Placing the bark at the base of the tree was significantly less effective and resulted in far fewer egg masses than the other two methods. The means for the older trees ranged from 6.5 for the Place Bark treatment to 57.3 and 80.5 woolly masses, respectively, for the Attach Bark and Suspend Bolt methods. The 2 year—old seedlings exhibited a mean height of 14.5 ± 0.69 cm and diameter of 3.11 ± 0.34 mm, whereas the measurements for the 7 year—old trees were 65.3 ± 0.91 cm and 16 ± 0.45 mm, respectively. The older trees were infested with five times more eggs/crawlers than the smaller trees; however, at the end of the test some of the larger trees averaged more than fifty times the number of adult adelgids. Infesting in the fall resulted in an average of 8.6 adults per tree versus 5.4 adults on trees in the summer. For both seasons, placing bark at the base of the seedling resulted in fewer adult adelgids on the seedling.

The standard error for the Suspend Bolt treatment was similar to those of the other two treatments (Tables 1 and 3). However, because the Suspend Bolt treatment is novel and offers less control over the number of adelgids applied, a look at the raw data is warranted. The number of woolly masses on 7 year—old trees from the Attach Bark treatment ranged from 0 to 183. The number from the Suspend Bolt treatment ranged, with one exception, from 33 to 219. The exception was one tree with a total of 445 woolly masses; this outlier was removed from the dataset and was not included in the analysis reported above.

### Study II: Density distribution of crawlers dropping from bolts

**Figure 1.**
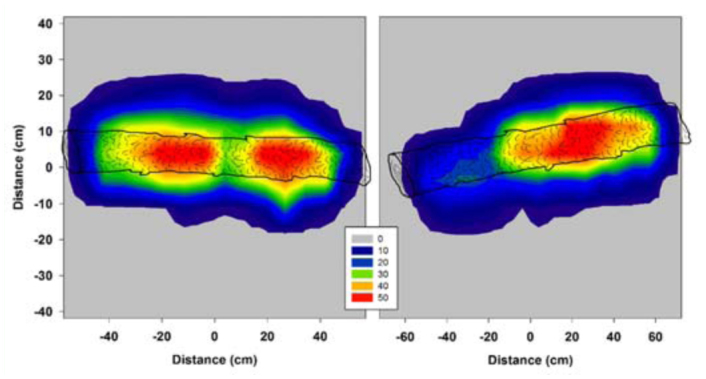
(Study II) *Adelges piceae* crawler density and distribution under the two logs. The image on the left reflects crawler densities under the 1.2 m log with little air flow and on the right the 1.5 m log in the air conditioned greenhouse space. The legend reflects the number of crawlers per square centimeter. The crawlers fell in higher densities directly under the logs, but they drifted an additional 10 cm on either side, providing good coverage within a 30 cm area. The differences in densities are a reflection of ‘hot spots’ of infestation where population densities were very high. High quality figures are available online.

Over 82,000 crawlers dropped from each of the bolts suspended over the paper grids in these studies. Over the entire grid, crawler abundance within each cell (58 cm^2^) ranged from 0 to over 3000 per cell or 0 to 50 crawlers per cm^2^ (Figure 1). Just under the bolt, crawler abundance ranged from 500 to over 3000 per cell (9 to 51 crawlers per cm^2^). The highest quantities of crawlers were found directly beneath the suspended logs, but the crawlers also drifted or fanned out away from the bolt as they fell. Hundreds of crawlers fell into each cell up to 10 cm on either side of the bolt, with the numbers rapidly dwindling in the cells approaching the opposite edges of the grid. Five hundred to one thousand eggs or crawlers have been suggested to achieve spot infestations on mature fir trees (Hollingsworth 1990), and successful infestations of Fraser fir seedlings have been achieved with 100 eggs (by attaching infested bark pieces) (Newton, unpublished data). Thus, a heavily infested bolt 10 cm in diameter could be expected to produce enough crawlers to infest fir seedlings or branches within a 30 cm diameter band underneath and on either side of the suspended bolt.

### Study III: Infestation of excised branches from 4 year—old Fraser fir and Veitch fir seedlings

After four weeks, the excised branches from both species were in good condition. Settled neosistentes were found on almost all of the branches close to the attached bark disc. After 7 weeks, the cuttings were still healthy and 9 Fraser fir branches contained adults with eggs. No *A. piceae* development was observed on the Veitch fir branches. After 9 weeks, the excised branches were still relatively healthy and the 10 Fraser fir branches had adults (total of 47) with eggs (total of 686). Fecundity ranged from 0–53 eggs per adult. Egg masses were located primarily at the base of buds, but also under old bud scales and at the base of needles along the cutting. On Veitch fir, only one branch had any sign of *A. piceae* development, with one adult (no eggs) and a single third instar. There were significant differences between species in all measured variables (Table 4). By the end of the 9 week period, most of the Veitch fir cuttings had broken bud, while the Fraser fir buds had not yet elongated.

**Figure 2.**
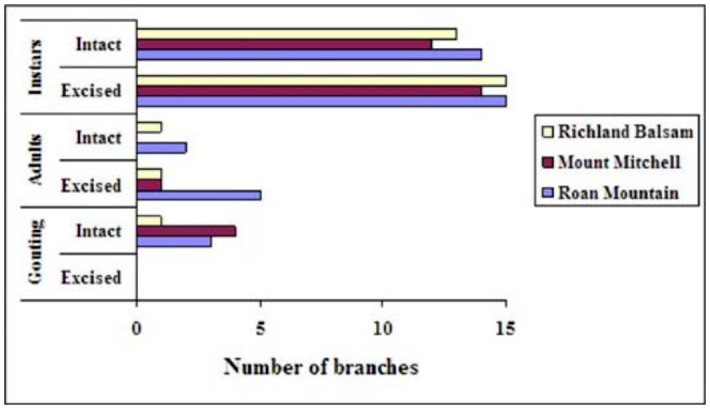
(Study IV) A comparison of intact versus excised branches from 4 year—old Fraser fir seedlings from three different seed sources. This figure presents the number of branches from each branch form (intact or excised) that contained settled first instars or adults, and also the number of branches within each form that responded to the presence of *Adelges piceae* through gouting (swelling at the site of infestation). Branches from the mature trees are not included here. High quality figures are available online.

### Study IV: Artificial infestation of intact and excised branches of 4 year—old Fraser fir seedlings and excised branches of 25— year—old Fraser fir

After 10 weeks, intact branches from all 15 seedlings were in good condition. Of the excised branches, 30 of the 45 branches taken from 4—year—old seedlings were in good condition; only two of the 15 branches from mature trees were in fair condition, and the remaining 13 branches were in poor condition. In comparing the intact and excised branches from the seedlings, all intact branches and all but one of the excised branches contained settled first instars (Figure 2). Three (3) intact and 9 excised branches produced adults; 2 of the excised branches (both from the Roan Mountain seed source) produced adults with eggs. Both source and form were significant for measured variables (Table 5). Trees from the Roan Mountain seed source produced significantly more adults than Mount Mitchell trees (Table 6). There were no source × form interactions, and source rankings for each measured variable were the same for intact and excised branches. There were more settled instars on the excised branches than on the intact branches. The intact branches responded to the presence of settled *A. piceae* instars in the form of gouting (swelling) at the site of infestation, and the excised branches exhibited no gouting response. Settled instars were found primarily at the base of buds, regardless of whether the infested bark discs had been placed near the bud or at the branch node.

A comparison between excised branches from 4 year—old seedlings and 25 year-old trees revealed no significant differences in the numbers of settled neosistentes or adults, but at the end of the trial, the material from the mature trees was in significantly poorer condition (Table 7). No gouting was observed on any of the excised branches.

## Discussion

Attaching bark and suspending infested bolts (Study I) are both effective techniques for artificially infesting fir trees with *A. piceae*. It is important to note that in this study each treatment was applied on three separate occasions to ensure a high number of crawlers. Thus, with the attach bark technique, each 7 year—old tree had 500 eggs applied three times for a total of 1500 eggs, and each 2 year—old tree had 100 eggs applied three times for a total of 300 eggs. Ideally, one application would provide sufficient numbers of adelgids to effect an infestation, but this is often difficult to achieve with the attach bark technique. Because the bark pieces must be very small to allow for a connection between the bark and the uninfested tree, there are limitations to the number of egg masses that can be introduced at one time. Additionally, the relative health of each egg clutch on infested source Fraser fir trees varies. With the exception of the earliest spring generation, there are multiple overlapping generations of *A. piceae* found on infested trees, and it is difficult to gauge how healthy a given population will be when the pieces are cut and applied to uninfested material.

The age effect noted in the infestation techniques study (I) was not surprising. The most obvious explanation lies in the size differences between the two age classes. Fewer eggs were applied to the younger trees and interception size may influence the Suspend Bolt technique. However, young seedlings in native stands of Fraser fir often appear resistant to *A. piceae* infestation (Hain 1988). In addition, although 1 and 2 year—old Fraser firs can be artificially infested with *A. piceae* (Newton, unpublished data), it is more difficult to accomplish in younger than in older trees. Resistance in these young seedlings may include both morphological and chemical mechanisms. *Adelges piceae* frequently settles underneath old bud scales, on lenticels, in cracks and crevices in bark (Balch 1952), and at the base of buds. In very young seedlings, the stems are smooth and there are few internodes. Attaching bark pieces is difficult because physical damage can occur to the delicate stems in the process of handling the bark, and there is less area for the bark to contact. Thus, crawlers that emerge may simply fall off the bark piece rather than crawling onto the seedling, and those that crawl from the bark pieces onto the tree may crawl off of it entirely. Additionally, young trees may produce chemicals that inhibit adelgid development.

A suspended bolt can provide thousands of crawlers. Bark pieces dry out in a matter of days whereas the bolt provides suitable habitat for continued adelgid development for up to two or three weeks after the tree is cut. Hence, crawlers are continually produced from a bolt over a longer period of time than from a piece of bark. In this study, fresh bolts were suspended once a week for three weeks over the treatment plots. Varying the placement of each bolt during the treatment period ensured a relatively even distribution of falling crawlers onto the trees below, and the successful infestation of trees throughout each plot provides evidence that this was effective. Cutting bark discs and attaching them to 40 uninfested trees took approximately 4 to 5 hours with two experienced technicians. Suspending the infested bolt was a simple process accomplished by tying each end to a frame; it took approximately 30 minutes with two technicians to suspend four bolts over 4 sets of 10 seedlings. The suspended bolt technique provides a method by which entire tables of uninfested seedlings or cuttings can be exposed to *A. piceae* with minimal time and effort, thus increasing the numbers of seedlings that can be utilized in future host resistance studies, and maximizing the value of material brought in from the field. This technique may be adapted to screening trials both large and small, provided a sufficient quantity of infested material is available.

The numbers of *A. piceae* crawlers that fall from suspended bolts of heavily infested Fraser fir, as indicated in Study II, are more than sufficient to produce an infestation in susceptible fir trees. Because crawler densities were highest within about a 30 cm diameter band underneath the suspended bolt, it is advisable to place the bolts about 30 cm apart to ensure good coverage. Additionally, because the infestation levels on a given log will naturally be greater in some areas than others, the numbers of crawlers falling onto the uninfested material will vary. Evenly infested seedlings or trees are desirable components within host resistance screening trials and care should be taken to ensure an even distribution pattern when utilizing the suspended bolt technique. Further, appropriate replication and randomization of genetic entities (species, families, clones, etc.) in resistance screening trials would also ameliorate variation problems. Improvements to the method could include fans to circulate air, thus widening the natural drift in the dispersal of crawlers, and rotating or moving the bolts to increase coverage and reduce over-application of crawlers under ‘hot spots’ of infestation. Even with the potential for variance in the numbers of falling crawlers, the suspend bolt technique appears to be more effective (on a tree—by—tree basis) for infesting the older (7 year—old) trees than the attach bark technique. With the attach bark technique, although great care was taken to attach bark pieces with an equal number of *A. piceae* egg masses to each tree, there were still a number of trees with zero woolly masses at the end of the study.

The early attempts to infest excised branches with *A. piceae* and maintain health of those branches for a period of time sufficient to allow development and reproduction were successful (Studies III and IV), and this technique is promising on a number of levels. It is encouraging that in each case, an entire generation of *A. piceae* was produced within a few weeks. In Study III, the vast majority of Fraser fir (10 of 12 cuttings) produced adults with eggs. The difference in infestation levels between Fraser and Veitch fir was expected because Veitch fir is known to exhibit marked resistance to *A. piceae* infestation (Mitchell 1966; Hall et al. 1971).

The results from Study IV (intact versus excised branches) provide some interesting insights, particularly the lack of gout production at the infestation site of excised branches, despite their higher frequency of settled instars. Gouting, the production of abnormal parenchyma cells (larger and in greater quantities than normal tissue) at the site of infestation, is a response to the presence of *A. piceae* by many fir species (e.g., Fraser fir, balsam fir). There appears to be a growth stimulating substance in *A. piceae* saliva that prompts this response in susceptible trees (Balch 1952). The saliva appears to contain a substance that induces similar effects as the auxin indolyl—3—acetic acid, and disturbs the normal hormonal balance within the tree (Balch et al. 1964). The absence of gouting in the excised branches may call into question the validity of utilizing this method as an early resistance screening tool, because here there appears to be a clear difference between the intact and excised branches. In an ideal situation there would be no difference between the two. Adelgids would find the excised branches (cuttings) no more or less attractive than intact tissue, would develop at the same rate and level of fecundity, and the cuttings would respond to the presence of adelgids as would a tree with roots. However, this ideal is not likely possible. Excised branches are placed under physiological stress and the absence of roots alters normal active growth, at least to the extent that the gouting response is inhibited. It appears that the roots are involved in the production of gout, but this is not clearly understood and has not been researched. Although it may be unreasonable to expect excised branch tips to serve as perfect surrogates for whole trees, this technique may be useful in helping to sort out the mechanisms of host resistance. Constitutive defenses may be present in excised branches while induced defenses may be slowed or not expressed at all.

## Conclusion

The suspended bolt technique mimics natural dispersal and appears to be effective in producing *A. piceae* infestations in a time— efficient manner. This technique can be utilized for large or small studies and can be adapted for infestations of other wind— dispersed insects. Bolts of heavily infested Fraser firs are capable of producing tens of thousands of *A. piceae* crawlers, and could be hung for a period of time over one set of trees and moved to another set of trees, thus economizing the number of bolts required for infestations. However, care should be taken to ensure an even distribution of crawlers. The time—honored technique of tying individual pieces of infested bark to trees is time consuming yet effective, and can be utilized in small studies or studies requiring infestation of specific areas of a tree rather than the entire tree.

Excised branches from two fir species spanning the range of resistance to *A. piceae* were successfully infested, and adults with eggs were produced on many of the Fraser fir specimens and almost none of the Veitch fir specimens. These results are consistent with what would be expected from infestation of a highly susceptible and a highly resistant species of fir. Infestation of intact and excised branches from trees of three Fraser fir seed sources revealed no differences between the two forms in the numbers of adult adelgids that had developed on each branch by the end of the study. The excised branches appear incapable of producing a gouting response to the presence of the adelgid, a marked difference from intact branches. Although this is an indicator that excised branches cannot serve as perfect surrogates for intact branches, it may help to further our understanding of defense mechanisms and physiological responses to the feeding of *A. piceae*. Lastly, excised branches taken from mature Fraser fir trees were shown to be capable of being infested, although the quality of the older specimens declined more rapidly than the younger material. The excised branch technique requires further study, but is promising for providing a nondestructive technique for initial host resistance screening trials.

**Table 1.**
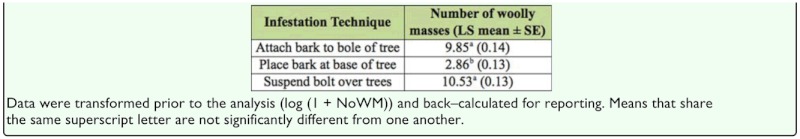
(Study I) LS mean (± SE) number of *Adelges piceae* woolly masses (NoWM) per tree by infestation technique.

**Table 2.**
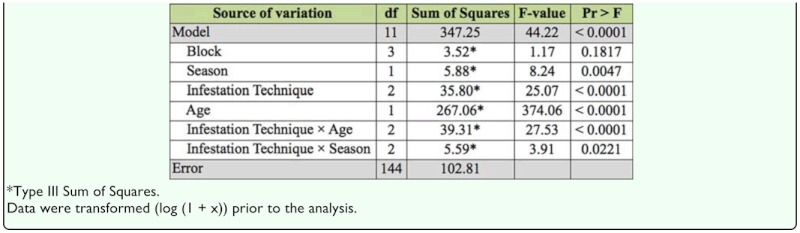
(Study I) Analysis of variance for the mean (LS mean) number of *Adelges piceae* woolly masses per tree.

**Table 3.**
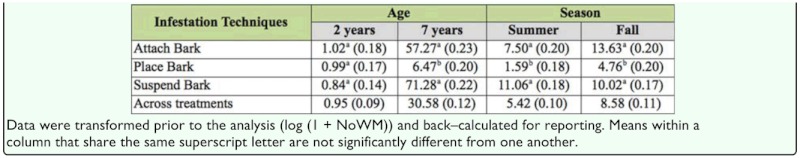
(Study I) LS mean (± SE) number of *Adelges piceae* woolly masses per tree, by age and season.

**Table 4.**
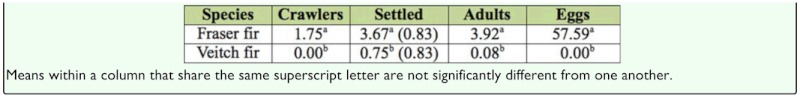
(Study III) LS means (± SE) for excised branches of Fraser and Veitch fir.

**Table 5.**
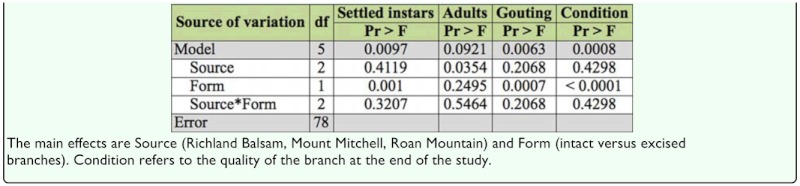
Study IV, 4 year—old seedlings only; intact versus excised branches. ANOVA *p*-values of the numbers of settled first instars, presence/absence of adults, presence/absence of gouting, and an estimate of condition.

**Table 6.**
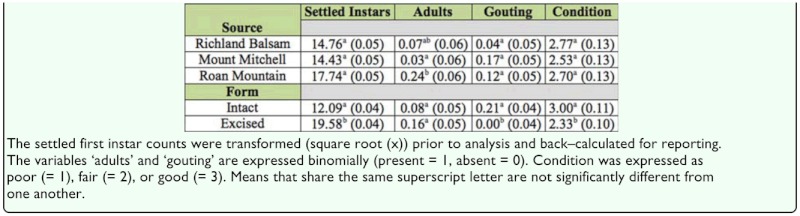
Study IV, 4 year—old seedlings only. LS means (± SE) for the main effects Source (Richland Balsam, Mount Mitchell, Roan Mountain) and Form (intact branch versus cutting).

**Table 7.**
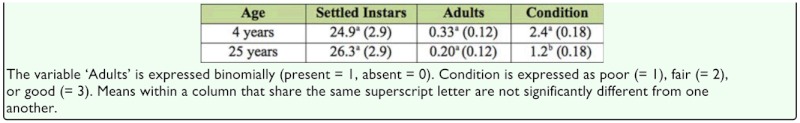
(Study IV) LS means (± SE) for excised branches of 4 year Roan Mountain seedlings (N = 15) and mature trees (N = 15).
